# The Utility of Infectious Disease Prevalence Studies to Inform Public Health Decision-Making in the Samoan Islands: A Systematic Review

**DOI:** 10.3390/tropicalmed10030071

**Published:** 2025-03-10

**Authors:** Beatris Mario Martin, Alison Brown, Filipina Amosa-Lei Sam, Aifili Tufa, Luis Furuya-Kanamori, Colleen L. Lau

**Affiliations:** 1 Centre for Clinical Research, Faculty of Health, Medicine, and Behavioural Sciences, The University of Queensland, Brisbane, QLD 4006, Australia; alison.brown@anu.edu.au (A.B.);; 2 Department of Pathology, Dunedin School of Medicine, University of Otago, Dunedin 9016, New Zealand; filipina.amosa@gmail.com; 3 American Samoa Health Department, Tafuna, Pago Pago, AS 96799, USA

**Keywords:** seroprevalence, serosurvey, infectious diseases, Samoa, American Samoa, surveillance

## Abstract

We conducted a systematic review of infectious disease (ID) prevalence studies in the Samoan Islands from 2000 to 2024 and aimed to synthesise the prevalence of IDs, the purpose of the studies, and the potential utility of survey results for informing public health decision-making. We searched five academic databases, the Western Pacific Region Index Medicus, and grey literature up until April 2024. English language publications of ID surveys in American Samoa and Samoa were included. Each study’s aim, design, and prevalence results were extracted and categorised by disease and data sources. We identified 46 publications reporting the prevalence of 15 different IDs; 42 publications (91%) reported data from 31 original surveys, of which three (9%) investigated the prevalence of multiple IDs. Twenty-eight (62%) publications primarily aimed to report prevalence to inform public health interventions. Samples from one survey, initially conducted for leptospirosis, were subsequently tested to confirm transmission, describe prevalence, and investigate risk factors for seven other diseases. We emphasise the valuable contribution of prevalence studies in supporting evidence-based public health interventions. The extensive prevalence studies in the Samoan Islands illustrate the need for adopting integrated multipathogen approaches to surveillance to reduce costs, document burden, and generate actionable insights to support evidence-based decisions to prevent, control, and eliminate infectious diseases.

## 1. Introduction

Infectious diseases are responsible for substantial morbidity and mortality across the Pacific Islands Countries and Territories (PICTs) [1,2]. Yet, accurate population-level estimates of the burden of infectious diseases (ID) are still limited in the region [3,4,5]. In the PICTs, higher vulnerability to extreme climate events due to geographic, climate, sociodemographic, and economic characteristics exacerbates health risks beyond socio-economic factors [2,6]. Events such as flooding, tsunamis, and progressively warmer days and nights, combined with limited resources for effective response, create favourable conditions for the transmission and (re-)emergence of ID, including the persistence of neglected tropical diseases (NTDs) and vaccine-preventable diseases (VPDs) [7]. In 2019, a measles outbreak affected multiple countries and territories in the Pacific, including Samoa, American Samoa, Fiji, and Tonga, revealing important immunity gaps in the region and susceptibility to outbreaks that can rapidly overload public health systems [8].

In this context, public health decision-making relies on robust populational-level health data across multiple administrative levels [9]. Understanding the prevalence and local risk factors for IDs can enhance the development of targeted interventions, thereby improving their impact.

To obtain robust population-level ID data, two approaches are frequently adopted: passive, routine disease surveillance (e.g., case notifications from hospital, laboratory, or syndromic surveillance systems), and prevalence surveys conducted on whole populations or sub-populations. Although routine surveillance data are relatively cost-effective and accessible [10], their quality can be limited by inconsistencies in healthcare provider reporting, the broad and non-specific nature of most febrile illnesses, and the restricted availability of diagnostic tests. On the other hand, population-wide prevalence surveys can more precisely measure populational-level immunity to diseases from infection or vaccination. By generating high-resolution data, prevalence surveys are frequently used to determine and monitor the extent of national disease burdens and their geographical distribution, investigate previously undetected or emerging pathogens, inform control or elimination activities, and screen population immunity to guide vaccination programmes. Prevalence studies can be used to collect data on risk factors for infection that can inform prevention, control, and elimination strategies [10]. Despite being extremely useful, these studies can be expensive and time-consuming to conduct, which makes frequent surveys impractical in resource-limited settings.

Our study aimed to review all prevalence surveys of ID in Samoa and American Samoa, synthesise the studies’ purposes, summarise the prevalence of ID, and describe the potential utility of survey results for informing public health decision-making.

## 2. Materials and Methods

This systematic review was reported following Preferred Reporting Items for Systematic Reviews and Meta-Analysis (PRISMA) guidelines (Supplementary Materials) [11]. This review was registered with the International Prospective Register of Systematic Reviews (PROSPERO) (CRD42023403740).

### 2.1. Search Strategy

A systematic search was undertaken on 17 May 2023 in PubMed, Embase, and CINAHL via EBSCOhost, Scopus, Web of Science, and Western Pacific Region Index Medicus-(WPRO) and included studies published after 1st January 2000. The search was updated on 25 April 2024. The key search terms were: ‘Samoa’, ‘American Samoa’, ‘communicable diseases’, ‘infectious’, ‘transmissible’, ‘viral’, ‘parasite’, ‘bacteria’, ‘epidemiology’, ‘prevalence’, and ‘surveillance’. The search terms were adapted to each database, and the full search strategy is available in Supplementary Materials. Google Scholar and grey literature, including the Samoa Ministry of Health website and the World Health Organization (WHO) Western Pacific Regional Office (WPRO) database, were also searched using key terms. The citation lists of included articles were hand-searched for additional relevant publications.

### 2.2. Inclusion and Exclusion Criteria

We included reports on the prevalence of ID classified as NTD, vector-borne diseases (VBDs), arboviruses, VPD, blood-borne viruses (BBVs) or zoonotic diseases, conducted in Samoa or American Samoa and published between 2000 and 2024, with the results restricted to publications in English.

We excluded incidence reports, as these can often be inaccurate due to underreporting and limitations of routine surveillance previously listed. Additionally, we excluded case reports, case series, and outbreak reports; those examining the prevalence of infection in vectors only (e.g., molecular xenomonitoring of mosquitoes); and publications that reported on simulation or prediction rather than the collection of primary data, such as modelling studies.

### 2.3. Screening Process

The titles and abstracts of retrieved publications were uploaded to Covidence (Veritas Health Innovation Ltd., Melbourne, VIC, Australia) [12] for screening. After the removal of duplicates, two authors (AB and BM) independently screened the titles and abstracts for studies that met the inclusion criteria. Full-text versions of all potentially relevant publications were retrieved and assessed for eligibility by the same authors. Disagreements were resolved by discussion. If it could not be resolved, a third researcher (LFK or CLL) was consulted.

### 2.4. Data Extraction

Data from the included publications were extracted and recorded in a Microsoft Excel (Microsoft Corporation, Redmond, WA, USA) [13] spreadsheet by AB and cross-checked by BMM. The extracted information included: general information about the publication (authors, title, and year of publication), study location, spatial scale, infectious disease(s) investigated, period of data collection, aims, sampling design, number of participants, type of specimen tested, and diagnostic test used, characteristics of the study population included (age and gender) and prevalence as reported in the publication (number of participants with positive results and total participants investigated).

### 2.5. Synthesis Methods

Publications included in this systematic review were aggregated by type of IDs reported (NTD, VPD, VBD, arboviruses, BBV, and zoonoses), by data source (surveys, national health reports, and summary publications) and by location (American Samoa and Samoa). Although some diseases could be classified into multiple categories (e.g., hepatitis B is both VPD and BBV), each disease was allocated to only one main category as decided by consensus among authors.

The aims of the publications were categorised into six groups: (1) to assess the evidence of ongoing transmission of an ID or identify the baseline prevalence conducted before a planned intervention, (2) to assess or monitor the prevalence or quantify transmission of known endemic IDs, (3) to identify hotspots or characterise spatial variation in seroprevalence, (4) to identify risk factors associated with high prevalence, (5) to assess the prevalence identified by a new (innovative) diagnostic, (6) to assess the impact of public health interventions (e.g., mass drug administration (MDA), vaccination, or counselling).

This study focused on providing a comprehensive description of publications on the prevalence of ID and the impact of results on public health decision-making. We included several types of publications, including peer-reviewed manuscripts, grey literature, and government reports. Meta-analysis was not conducted because of the limited number of studies for each disease, and study design varied between studies. Thus, a quality assessment of the publications was not conducted.

## 3. Results

### 3.1. Yield of Search Strategy

**Figure 1:**
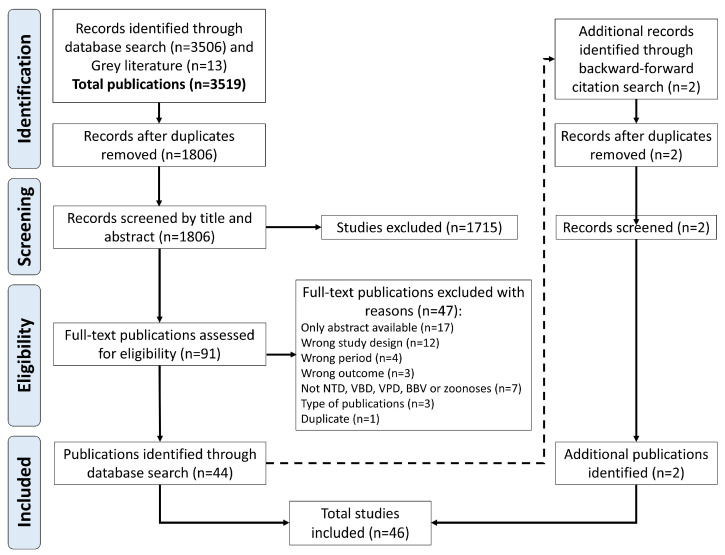
Preferred Reporting Items for Systematic Reviews and Meta-Analyses (PRISMA) flow chart showing search and selection of studies procedure. BBV: blood-borne viruses, NTD: neglected tropical diseases, VBD: vector-borne diseases, VPD: vaccine-preventable diseases.

The initial search strategy yielded 3519 records; after excluding duplicates, 1806 records were retained and screened by title and abstract, and 1715 studies were excluded. After screening, 91 articles went through full-text review, and 44 of these met the inclusion criteria. Two articles identified through hand searching the bibliographies of retrieved publications met inclusion criteria and were included in this review, resulting in 46 publications being included for analysis (Figure 1).

### 3.2. Characteristics of Included Publications

#### 3.2.1. Data Source

Of the 46 publications included in this systematic review, 42 (91.3%) reported on data obtained from surveys (Supplementary Table S1). Thirty-one original surveys were identified across these publications. The primary data source of four publications (8.7%) were not a survey or were not limited to surveys; two (4.3%) were national health reports of population screening activities, and two (4.3%) were publications summarising lymphatic filariasis (LF) prevalence data from multiple sources (surveys, government reports, and previously unpublished data).

#### 3.2.2. Setting

Among the 31 original surveys identified across the included publications, 20 (64.5%) were conducted in American Samoa and 11 (35.5%) in Samoa. All national reports were from Samoa (Table 1). Of the two publications summarising LF prevalence, one included data from both American Samoa and Samoa [14], and the other only from Samoa [15].

#### 3.2.3. Target Population

Twelve surveys (38.7%) were community-based, six (19.4%) were school-based, five (16.1%) were based on pre-existing healthcare programmes, four (12.9%) were conducted in targeted villages or communities with previously known high prevalence of a disease, two (6.5%) targeted population based on occupation, and two (6.5%) did not report the population included. Five surveys (16.1%) were conducted exclusively among children (age < 18 years), nine (29.0%) were among adults, and 13 (41.9%) included all ages (from <1 to 94 years). In three surveys (9.7%), participants’ age was not reported.

#### 3.2.4. Infectious Diseases Reported

Publications reported on 15 different IDs (Table 1). The most frequent ID studied was LF, with 25 publications (54.3%), of which 16 were from American Samoa (64.0%), eight from Samoa (32.0%), and one from both (4.0%). Four publications (8.7%) reported the prevalence of Human Immunodeficiency Virus (HIV), all from Samoa. Another four publications (8.7%) reported the prevalence of hepatitis B virus, one from American Samoa and three from Samoa. Three publications (6.5%) reported hepatitis C virus prevalence, two from Samoa and one from both American Samoa and Samoa. Three publications (6.5%) reported leptospirosis prevalence and two (4.35%) dengue (DENV) prevalence, all from American Samoa. Two publications (4.3%) reported on scabies clinical prevalence, both in Samoa. Zika virus (ZIKV), Ross River virus (RRV), varicella zoster virus (VZV), human papillomavirus (HPV), and rickettsia prevalence were reported in one publication each (2.2%), all from American Samoa.

#### 3.2.5. Aims of Studies

**Figure 2:**
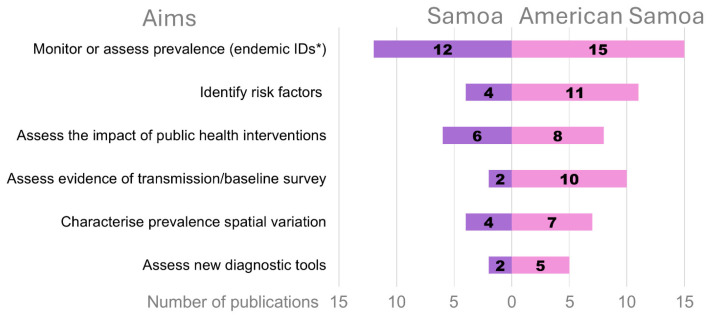
Aims of the publications reporting on the prevalence of neglected tropical diseases, vector-borne diseases, vaccine-preventable diseases, blood-borne viruses and zoonotic diseases in American Samoa and Samoa, 2000–2023. * IDs: Infectious diseases.

Twenty-four publications aimed to assess or monitor the prevalence or quantify ongoing transmission of a known endemic ID (52.2%). Of these, 14 (58.3%) were from American Samoa, 11 (45.8%) were from Samoa, and one (4.2%) was from both locations. Fifteen publications (32.6%) aimed at identifying risk factors associated with prevalence or transmission, 11 (73.3%) in American Samoa and four (26.7%) in Samoa. Fourteen publications (30.4%) aimed to assess the impact of a public health intervention, eight (57.1%) from American Samoa and six (42.9%) from Samoa. All aims are summarised in Figure 2.

### 3.3. Disease Prevalence

The prevalence of each of the 15 diseases in American Samoa and Samoa between 2000 and 2024, as reported by included publications, is presented in Supplementary Material Table S2 (LF) and Supplementary Material Table S3 (all other diseases).

#### 3.3.1. American Samoa

LF prevalence was assessed mainly by rapid antigen tests (BinaxNOW Immunochromatographic Tests (ICTs) or Alere/Abbott Filariasis Test Strips (FTS)), used in all surveys originally designed to investigate LF antigen prevalence. Other serodiagnostic tools for LF included the Og4C3 antigen and the Bm14, Bm33, and Wb123 antibodies (Supplementary Table S3).

There was an overall trend of decline in the prevalence of LF antigenaemia, from 11.5% (95%CI not reported) in community sentinel sites in 2001 [17], to 2.7% (95%CI not reported) in a community-based survey conducted in 2019 [26]. This period reflects the rollout of the Pacific Programme to Eliminate LF (PacELF) and efforts to eliminate LF from the territory using MDA from 2000. The lowest prevalence reported was 0.1% (95%CI 0.0–0.7) in school-age children (Transmission Assessment Survey-2, TAS-2) in 2015 [21]. In 2016, a resurgence of LF was noted with an estimated prevalence of 0.8% (95%CI 0.4–1.5%) in schools (TAS-3) [22,27,49], and 4.1–5.1% in community surveys [22,23,28,29,32]. The adjusted prevalence from TAS-3 was above the target threshold of 1% for stopping MDA [17,22,32].

Leptospirosis seroprevalence was assessed through microscopic agglutination testing (MAT). The results from two surveys conducted in 2004 [41] and 2010 [40] suggest high endemicity, with 17.0% and 15.5% prevalence, respectively. The three predominant serovars (*Leptospira interrogans* serovars (1) Hebdomadis, (2) Pohnpei (LT 751), and (3) LT 1163) identified in the 2010 survey differed from those identified in the 2004 survey and were not previously known to occur in American Samoa [41]. Additionally, the three serovars differed in geographical distribution and drivers of transmission. The seroprevalence of the serovar Hebdomadis was associated with animal exposure, while serovars Pohnpei (LT 751) and LT 1163 were associated with recreational exposure.

In the subsets of data collected through this survey conducted in American Samoa in 2010, originally designed to study leptospirosis, the presence of immunoglobulin G class (IgG) antibodies against arboviruses (DENV, RRV) was determined by enzyme-linked immunoassay (ELISA), and against rickettsia (*Rickettsia felis*, *Rickettsia typhi*, *Rickettsia conorii*, *Coxiella burnetii*, *Bartonella henselae*, *Bartonella quintana*, *Bartonella clarridgeiae*, and *Ehrlichia chaffeensis*) were determined by immunofluorescence assay (IFA). The results were reported across three publications; in 2013, Duncombe et al. [33] reported that antibodies against DENV were present in 95.6% of the participants tested; in 2016, Lau et al. [43] reported the absence of serological evidence of rickettsial, bartonella, ehrlichia, and *C. burnetii* species; and in 2017, Lau et al. [34] reported an antibody prevalence of 74.1% against RRV, strongly suggesting endemic circulation. Another survey was conducted in 2017 to investigate dengue prevalence in the territory [36]. This survey was conducted among household members of laboratory-confirmed dengue cases and found a 3.1% prevalence of acute dengue infection (either real-time polymerase chain reaction (RT-PCR) or anti-DENV Immunoglobulin M class antibodies (IgM ELISA)).

The prevalence of soil-transmitted helminth infections and antibodies to ZIKV, VZV, and hepatitis B and C were reported in only one publication each and results are shown in Supplementary Table S4.

#### 3.3.2. Samoa

LF seroprevalence was assessed mainly by antigen (ICT or FTS), while antibodies (Bm14, Bm33, or Wb123) were less frequently used in Samoa compared to American Samoa (Supplementary Table S3). Overall, LF antigen prevalence decreased over the years, from 8.1% (95%CI not reported) in 2000 [14] to 4.7% (4.0–5.6%) in 2019 [50] in community-based surveys. However, the antigen prevalence reported in Samoa did not show a linear decline. Between 2004 and 2013, the national level antigen prevalence was low, from 1.1% [15,44] to 0.7% [15]. Yet, surveys conducted in 2008 in specific communities showed great variability in antigen prevalence across the country, from 1.6% (95%CI 0.7–3.2) to 14.6% (95%CI 11.9–17.6%) [15,46,47]. More recently, a sustained increase at the national level has been identified, with a reported prevalence of 3.9% in 2017 (TAS-2), 4.3% in 2018 (population representative cluster survey), and 4.7% in 2019 (stratified clustered survey) [15,48,49,50].

Scabies prevalence was assessed in three surveys by clinical examination. Two surveys were conducted in 2018, and one in 2019. These studies demonstrated a higher prevalence in school-age children in rural areas of 14.4% (95%CI 12.1–17.0) [37] compared to the nationwide prevalence of 3.0% (95%CI 2.5–3.6) in 2018 and 4.2% (95%CI 3.6–4.8) in 2019 [52].

The prevalence of blood-borne viruses (BBVs) was examined in surveys conducted between 2000 and 2008, before screening programmes were implemented [53,54,55,56,59], and from national surveillance activities via prenatal, blood donor and immigration screening clinics from 2017 [57,58]. Reports from 2000, 2004 to 2005, and 2008 found no positive cases of HIV among sampled pregnant women attending routine prenatal care [53,54,56]. No cases of HIV were identified in 2004–2005 among a sampled population attending an STI-HIV/AIDS clinic [54].

In 2008, hepatitis B surface antigen (HBsAg) prevalence among women attending prenatal clinics was 10.7% (95%CI 7.6–15.0%) [55]. National health reports refer to blood screening tests against hepatitis B and C, and HIV, without identifying the specific diagnostic tests used. HBsAg prevalence from prenatal screening clinics in 2017, and 2018 was 1.4% and 1.2%, respectively [57,58]. Among immigrant workers (countries of origin not reported), hepatitis B prevalence (specific test not reported) was 2.3% in 1737 people tested in 2017, and 2.1% in 1813 people tested in 2018 [57,58]. Among blood donors, hepatitis B prevalence was 3.1% in 1382 donors tested in 2017, and 2.3% in 3041 donors tested in 2018 [57,58].

A survey conducted in 2002 identified a 0.08% prevalence of hepatitis C among the general population [59]. In 2017, there were no positive cases of hepatitis C in all the 3012 women tested at the prenatal clinic and in 2018, two cases were identified among 4266 women tested at the prenatal clinic (0.05%). Among immigrant workers, hepatitis C prevalence was 0.06% in 1609 people tested in 2017 and no cases were identified in 2018 among the 1608 people tested. Among blood donors, hepatitis C prevalence was 0.07% in 1379 people tested in 2017 and 0.14% in 2958 people tested in 2018, respectively [57,58].

### 3.4. International Collaborations

Of the 41 peer-reviewed publications, the lead authors were from Australia (28; 68.3%), U.S. (12; 29.3%), and New Zealand (1; 2.4%). Of the 28 publications on American Samoa, 17 (60.7%) were led by Australia, and 11 (39.3%) by U.S. Of the 12 publications on Samoa, 11 (91.7%) were led by Australia and one (8.3%) by New Zealand. One paper that included both American Samoa and Samoa was led by U.S. Studies led by U.S. were predominantly supported by U.S. CDC, while those led by Australia were supported by researchers from academic institutions.

## 4. Discussion

This systematic review found 31 original surveys that examined the prevalence of 15 different IDs in American Samoa and Samoa between 2000 and 2024. These publications provided invaluable information regarding the prevalence, distribution and drivers of transmission for LF, HIV, hepatitis B and C, leptospirosis, arboviruses, scabies, HPV, varicella, and rickettsia. The studies found that despite efforts to eliminate LF, this NTD persists in both locations. In American Samoa, there was evidence of endemic transmission of dengue, RRV, and leptospirosis, but there have been no publications on surveys for these diseases in Samoa. Surveys in Samoa found a high prevalence of scabies, disproportionately affecting primarily school-aged children living in rural communities, but no surveys have been conducted in American Samoa.

The predominance of LF publications could be explained by the overlap of the years covered by this systematic review with the launch and implementation of the PacELF [14,15,60]. The programme might have contributed to a growing interest in LF prevalence in the region, leading to funding for multiple programmatic surveys and research studies. It is important to note that the two most recent surveys, originally designed to study LF in Samoa, included a clinical investigation of scabies and impetigo [48,52], suggesting the feasibility of implementing multi-disease surveys that combined clinical examination with serological testing.

During our study period, a larger number of surveys were conducted in American Samoa (supported by both the U.S. and Australia) than in Samoa (supported almost entirely by Australia). The higher proportion of publications led by Australia in both locations is likely to be a result of the combination of geographic proximity to the Pacific Islands, and strong regional networks and collaborations. It is important to note that the publications included in this systematic review might not represent the full extent of surveillance conducted in each location, as some national health reports might not be publicly available.

The diseases targeted varied between the two locations, with American Samoa presenting a wider range of infections and pathogens investigated. Of the non-LF publications in American Samoa (n = 11), almost half (n = 5) used samples from the same survey conducted in 2010 for leptospirosis (led by Australia) [33,34,40,41,43], with publications from 2013 to 2017 demonstrating the value for seroprevalence studies and serum banks for surveillance of multiple diseases. If multiplex bead assays were available for this survey, the results investigating multiple additional IDs could have been generated in a more timely manner and at a much lower cost. Conversely, in Samoa, the majority of the non-LF publications were from the Ministry of Health and focused on BBV screening, typically conducted using point-of-care and rapid tests [57,58]. Given the need for cost-effective public health interventions in resource-limited settings, existing surveillance programmes could be used as an opportunity to test for other diseases targeted by control and elimination programmes. Additionally, when appropriate, including BBVs in these surveys could facilitate testing populations that are frequently excluded from routine screening programmes.

The publications included in this systematic review reported a wide range of aims and diseases, and their results can and have been used to better inform a range of public health interventions. Studies that aimed to assess evidence of transmission or identify baseline prevalence contributed to the establishment of strategies to improve diagnoses (e.g., routine screening of hepatitis B and C in the prenatal population and blood donors [54,57,58,59]), and provided evidence to justify the need of a regional integrated surveillance approach to prevent a new outbreak (e.g., high prevalence of RRV in American Samoa [34]).

Studies aiming to quantify ongoing transmission generated information that helped identify a timeline to discontinue screening of asymptomatic pregnant women for Zika infection [35] and identified the need to maintain and intensify MDA for LF [22,23,29,32,38]. Studies reporting this aim also contributed to identifying high-risk groups to prioritise for varicella and hepatitis B vaccination, [37,39] and identified communities for more intensive LF surveillance [52]. By identifying risk factors associated with ID prevalence, authors were able to suggest targeted interventions that could improve transmission control, such as mosquito surveillance [33], or measures to block mosquito access to septic tanks [36] to prevent dengue transmission, infrastructure improvement (e.g., access to safe water and sanitation) to prevent STHs [37]), and piggery management (e.g., proper drainage and greater distance between piggeries and households [40]) to prevent leptospirosis transmission. In the context of the Global Programme to Eliminate LF, and with more countries progressing toward LF elimination, the assessment of new diagnostic tools (i.e., anti-filarial antibodies) to monitor LF transmission contributed to identifying high-risk areas of transmission and early signals of resurgence [18,19,25,45,46]. These findings will also be used to inform post-validation surveillance strategies in PICTs and other previously LF-endemic countries.

In the 20 years covered by this review, the number of studies for most diseases was small. This limited our ability to conduct country- and disease-specific analyses of trends, except for LF. The resources (cost, staff time, and technical expertise) required to conduct surveys likely impacted the number and type of IDs targeted, with a predominance of studies examining IDs that are marked for elimination (such as LF) or have well-funded global programmes (such as HIV) [61]. The number and quality of retrieved publications highlight the great efforts and resources that have been invested into generating high-quality population-level prevalence data to inform action, and confirm the high value that local public health decision-makers put into such data.

Most (28; 90%) of the surveys included in our study focused on a single disease. However, several publications [56,57] in recent years have noted the benefit of combining survey efforts to address multiple IDs simultaneously. Moving beyond a vertical, disease-specific approach, integrated surveys can be embedded within coordinated, horizontal public health frameworks that leverage shared resources, tools, and methodologies. Such approaches have the potential to create opportunities to monitor multiple diseases concurrently [62] and strengthen health system capacity by fostering cross-disease collaboration, data-sharing, and cost-saving. Such integrated strategies align with broader efforts to shift from fragmented, disease-specific programmes toward a more comprehensive and cost-effective public health surveillance system [62,63].

As the number of retrieved publications suggests, there has been extensive monitoring of IDs in the Samoan Islands through seroprevalence studies. If the same number of surveys was conducted using an integrated multi-pathogen approach, a review like this one could provide a much more comprehensive analysis of the trend in the prevalence of NTDs, VBDs, VPDs, and BBVs over time, while assessing the impact of public health interventions such as MDA or vaccination programmes.

This study emphasises the invaluable contribution of prevalence studies in supporting evidence-based public health decision-making. Conducting integrated, multi-disease prevalence surveys can help identify infectious diseases and populations to be prioritised in the allocation of resources. Additionally, there is a need for accessible, acceptable, and affordable diagnostics. Integrated serological surveillance, which involves detecting multiple antigen or antibody targets simultaneously, can be a relatively low-cost, efficient, and effective method for identifying population-level burden or immunity to a wide range of pathogens.

## Figures and Tables

**Table 1:** Characteristics of publications reporting the prevalence of neglected tropical diseases, vaccine-preventable diseases, vector-borne diseases, blood-borne viruses, and zoonoses in American Samoa and Samoa, 2000–2024.

First Author, Year	Disease	NTD	VBD	Arboviruses	VPD	BBV	Zoonoses	Aims ^a^	Sample Size	Age (Years)	Ref.
American Samoa											
Hughes, 2004	STH	●						1	60	5–12	[16]
Liang, 2008	LF	●	●					2, 6	1024	≥5	[17]
									917	≥5	
									1371	≥5	
Chu, 2013	LF	●	●					6	949	5–10	[18]
Lau, 2014	LF	●	●					2, 3, 5, 6	806	17–87	[19]
Lau, 2016a	LF	●	●					2, 5	376	17–87	[20]
Won, 2018	LF	●	●					2	937	5–10	[21]
									768	NR ^i^	
Sheel, 2018	LF	●	●					2, 3, 6	1143	5–10	[22]
									2496	8–93	
Lau, 2020a	LF	●	●					2, 4, 6	1143	5–10	[23]
									2496	8–93	
Graves, 2020	LF	●	●					4	670	≥5	[24]
Mladonicky, 2009	LF	●	●					2, 5	569	≥5	[25]
Hast, 2020	LF	●	●					2, 6	2081	≥5	[26]
Restrepo, 2022	LF	●	●					2, 5	937	5–10	[27]
									768	NR ^i^	
									1143	5–10	
Lemin, 2022	LF	●	●					4	2671	8–93	[28]
Wangdi, 2022	LF	●	●					2, 3, 5	2671	8–93	[29]
Lau, 2017a	LF	●	●					2, 3	1132	≥5	[30]
Coutts, 2017	LF	●	●					3, 4, 6	1881	≥2	[31]
									807	18–87	
Restrepo, 2023	LF	●	●					3, 4	2671	8–93	[32]
Duncombe, 2013	Dengue		●	●	●			1	794	18–87	[33]
Lau, 2017b	RRV		●	●				1, 4	196	18–87	[34]
Hancock, 2017	Zika		●	●				2	277	NR	[35]
Sharp, 2023	Dengue		●	●	●			2, 4	226	≤94	[36]
Mahamud, 2014	VZV				●			1	723	4–35	[37]
Hernandez, 2013	HPV				●			1	211	18–82	[38]
Ly, 2014	Hep B				●	●		1, 4, 6	231	19–71	[39]
Lau, 2012a	Leptospirosis						●	2, 4	807	18–87	[40]
Lau, 2012b	Leptospirosis						●	1, 3, 4	341	18–86	[41]
									807	18–87	
Winger, 2004	Leptospirosis						●	1, 4	341	8–86	[42]
Lau, 2016b	*Rickettsia* spp., *Bartonella* spp., *Ehrlichia* spp., *Coxiella burnetii*						●	1	197	18–87	[43]
**Samoa**											
Huppatz, 2009	LF	●	●					8	12,719	NR	[44]
Joseph, 2011c	LF	●	●					3, 5	2474	2–92	[45]
Joseph, 2011b	LF	●	●					2, 3	2474	2–92	[46]
Joseph, 2011a	LF	●	●					2, 5, 6	6648	≥5	[47]
Willis, 2020	LF	●	●					6	4213	≥2	[48]
Lau, 2020b	LF	●	●					2, 3, 4, 6	3940	≥5	[49]
Mayfield, 2020	LF	●	●					2, 3, 6	2322	≥5	[50]
									2594	≥5	
Graves, 2021	LF	●	●					8	NA		[15]
Taiaroa, 2021	Scabies	●						2, 4	833	5–15	[51]
Willis, 2023	Scabies	●						2, 4, 6	2868	0–75	[52]
									2796	0–75	
Sullivan, 2004	HIV					●		1, 6	441	15–48	[53]
MoH/WHO, 2006	HIV					●		2	299	15–44	[54]
									101	16–43	
MoH/WHO, 2008	Hep B, HIV				●	●		2	298	15–49	[55]
Cliffe, 2008	HIV					●		2, 4	119	15–44	[56]
MoH, 2018	HIV, Hep B, Hep C				●	●		2	Varied	NR	[57]
MoH, 2019	HIV, Hep B, Hep C				●	●		2	Varied	NR	[58]
**American Samoa and Samoa**											
WHO, 2006	LF	●	●					2	NA	NR	[14]
Armstrong, 2006	Hep C					●		1	1359	NR	[59]
									1289	NR	

STH: soil-transmitted helminthiasis, LF: lymphatic filariasis, RRV: Ross River viruses, VZV: varicella-zoster viruses, HPV: human papillomavirus, HIV: human immunodeficiency virus. ^a^ Aims: (1) to assess the baseline prevalence or prevalence before an intervention was implemented, (2) to assess or monitor the prevalence of a known endemic infection or quantify ongoing transmission, (3) to identify hotspots or characterise spatial variation in seroprevalence, (4) to identify risk factors associated with prevalence or transmission, (5) to assess the prevalence identified by a new (innovative) diagnostic tools, and (6) to assess the impact of public health interventions, e.g., mass drug administration, vaccines, health counselling. NA: Not available, summary studies included multiple years, and the final total population was not reported. ^i^—mean age 7 years old. NR: not reported.

## Data Availability

The authors confirm that the data supporting the findings of this study, including search terms and number of publications retrieved, are available in the Supplementary Materials.

## Supplementary Materials

The following supporting information can be downloaded at: https://www.mdpi.com/article/10.3390/tropicalmed10030071/s1, S1. Methods: S1.1 Search strategy, S2. Results: S2.1 Publications included in the systematic review: Supplementary Table S1. Characteristics of surveys conducted in the Samoan Islands between 2000 and 2020 by year; Supplementary Table S2. Publications included in the systematic review reporting data from sources other than surveys (national reports and summary of multiple studies), S2.2 Reported Prevalence: Supplementary Table S3. Samoan Islands’ lymphatic filariasis prevalence in publications, between 2000 and 2020 by year; Supplementary Table S4. Samoan Islands’ NTDs, VPDs, BVDs, BBVs, and zoonoses prevalence in publications, between 2000 and 2020 by year.

## Abbreviations

The following abbreviations are used in this manuscript:

ID	Infectious diseases
95%CI	95% confidence interval
AIDS	Acquired immunodeficiency syndrome
BBV	blood-borne viruses
DENV	Dengue virus
ELISA	enzyme-linked immunoassay
FTS	Filariasis Test Strips
HBsAg	Hepatitis B surface antigen
HBV	Hepatitis B virus
HCV	Hepatitis C virus
HIV	Human immunodeficiency virus
HPV	Human papillomavirus
ICT	Immunochromatographic Tests
IgG	Immunoglobulin G
IgM	Immunoglobulin M
LF	Lymphatic filariasis
MAD	Mass-drug administration
MAT	Microscopic agglutination test
NA	Not available
NTDs	Neglected tropical diseases
PacELF	Pacific Programme to Eliminate LF
PICTs	Pacific Islands Countries and territories
PRISMA	Preferred Reporting Items for Systematic Reviews and Meta-Analysis
PROSPERO	Prospective Register of Systematic Reviews
RRV	Ross-river viruses
RT-PCR	Realtime polymerase chain reaction
STI	Sexually transmitted infections
TAS	Transmission Assessment Survey
VBDs	vector-borne diseases
VPD	Vaccine-preventable diseases
VZV	varicella-zoster viruses
WHO	World Health Organization
WPRO	Western Pacific Regional Office
