# Supplementary material for: The Utility of Infectious Disease Prevalence Studies to Inform Public Health Decision-Making in the Samoan Islands: A Systematic Review
Source: Trop Med Infect Dis. 2025 Mar 10;10(3):71. doi: 10.3390/tropicalmed10030071 (PMC11945429; doi:10.3390/tropicalmed10030071)
Supplement: Supplementary file 1 [file tropicalmed-10-00071-s001.zip › tropicalmed-3480517-supplementary.pdf]

Supplementary Materials

The utility of infectious disease prevalence studies to inform public health decision-making in the Samoan Islands: A systematic review

Beatris Mario Martin[1]*, Alison Brown[1], Filipina Amosa-Lei Sam[2], Aifili Tufa[3], Luis Furuya-Kanamori[1], Colleen L Lau[1].

1- Centre for Clinical Research, Faculty of Health, Medicine, and Behavioural Sciences, The University of Queensland, Brisbane, Australia.

2- Department of Pathology, Dunedin School of Medicine, University of Otago. Dunedin, New Zealand

3- American Samoa Health Department, Tafuna, American Samoa*Corresponding author

Content:

1. **Methods:**
   a. **Search Strategy**

2. **Results**
   a. **Publications included in the systematic review**
   b. **Reported Prevalence**

3. **PRISMA checklist**

S1. Methods:

S1.1. Search Strategy

	Search terms	Number of publications retrieved 17/05/2023	25/04/2024[1]
PubMed	("Samoa"[Mesh] OR "American Samoa"[Mesh] OR "independent state of samoa"[MeSH Terms] OR samoa*) AND ("communicable diseases"[MeSH Terms] OR"communicable disease*"[Tiab] OR "endemic diseases"[MeSH Terms] OR endemic[tiab] OR "infection*"[Tiab] OR "infections"[MeSH Terms] OR "Infectious"[Tiab] OR "contagious disease*"[tiab] OR "transmissible disease*"[tiab] OR "viral"[tiab] OR "virus"[tiab] OR "parasit*"[tiab] OR "bacteria*"[tiab] OR "Epidemiology"[Mesh] OR "epidemiology"[Subheading] OR "epidemiolog*"[tiab] OR "surveill*"[tiab] OR "morbidity"[MeSH Terms] OR morbidity[tiab] OR Prevalence[mesh] OR prevalen*[tiab] OR endemic[tiab] OR monitor*[ti]) AND ((2000:3000/12/12[pdat]) AND (english[Filter]))	780	75
Embase.com	('samoa'/exp OR 'samoan islands'/exp OR 'american samoa'/exp OR samoa*) AND ('communicable disease'/exp OR 'communicable disease*':ti,ab OR 'endemic disease'/exp OR 'infection*':ti,ab OR 'infection'/exp OR 'infectious':ti,ab OR 'contagious disease*':ti,ab OR 'transmissible disease*':ti,ab OR 'viral':ti,ab OR 'virus':ti,ab OR 'parasit*':ti,ab OR 'bacteria*':ti,ab OR 'epidemiology'/exp OR 'epidemiolog*':ti,ab OR 'surveill*':ti,ab OR 'morbidity'/exp OR morbidity:ti,ab OR 'prevalence'/exp OR prevalen*:ti,ab OR endemic:ti,ab OR monitor*:ti) AND [english]/lim AND [embase]/lim AND [2000-2023]/py	73	37
CINAHL via EBSCOhost	(MH "American Samoa" OR MH "Independent State of Samoa" OR MH "Samoa" OR samoa*) AND (MH "Communicable Diseases+" OR "communicable disease*" OR endemic OR "infection*" OR MH "Infection+" OR "Infectious" OR "contagious disease*" OR "transmissible disease*" OR "viral" OR "virus" OR "parasit*" OR "bacteria*" OR MH "Epidemiology+" OR "epidemiolog*" OR "surveill*" OR MH "Morbidity+" OR morbidity OR MH "Prevalence" OR prevalen* OR endemic OR TI monitor*)	261	3
Scopus	(TITLE-ABS (samoa*)) AND (TITLE-ABS ("communicable disease*" OR endemic OR "infection*" OR "Infectious" OR "contagio us disease*" OR "transmissible disease*" OR "viral" OR "virus" OR "parasit*" OR "bacteria*" OR "e pidemiolog*" OR "surveill*" OR morbidity OR prevalen* OR endemic) OR TITLE (monitor*)) AND PUBYEAR > 1999 AND (LIMIT-TO (LANGUAGE , "English"))	530	2
Web of Science	#1: TS=("communicable disease*" OR endemic OR "infection*" OR "Infectious" OR "contagious disease*" OR "transmissible disease*" OR "viral" OR "virus" OR "parasit*" OR "bacteria*" OR "epidemiolog*" OR "surveill*" OR morbidity OR prevalen* OR endemic) OR TI=(monitor*)-<br>#2: TS=(samoa*)<br>#3: #2 AND #1<br>#4: #2 AND #1 and English (Languages)<br>#5: (#2 AND #1) AND (LA==("ENGLISH"))	588	241
WPRO[2]	Samoa*	12	0

[1] Number of publications added to the initial search. [2]WPRO: Western Pacific Region Index Medicus – https://www.globalindexmedicus.net/biblioteca/wprim

S2. Results

S2.1. Publications included in the systematic review

Supplementary Table S1. Characteristics of surveys conducted in the Samoan islands between 2000–2024 by year.

Year conducted	Study population			Study design		Publications			
	**Geographical level**	**Target groups**	**Age (years)**	**Sampling design**	**Sample size**	**Ref**	**First author, year**	**Disease**	**Sample size reported**
**America Samoa**									
2001-2002	National	School based survey	5 –12	Convenience	60	[16]	Hughes, 2004.	STH	60
2002	NR	NR	NR	NR	1359	[59]	Armstrong, 2006	HCV	1359
2001	National	Sentinel villages	5–≥60	Convenience	1024	[17]	Liang, 2008	LF	1024
2003	National	Sentinel villages	5–≥60	Convenience	917	[17]	Liang, 2008	LF	917
2004	National	Population representative	18–86	Random	341	[42]	Winger, 2004	Leptospirosis	341
						[41]	Lau, 2012b	Leptospirosis	341
2006	National	Sentinel and spot -check villages	5–≥60	Convenience	1371	[17]	Liang, 2008	LF	1371
2006	National	Community based survey	≥5	Census	569	[25]	Mladonicky, 2009	LF	569
2007	National	Community based survey (C-survey)	≥2	Random	1881	[31]	Coutts, 2017	LF	1881
2008-2009	Sub-national	Community and Hospital-based	18–82	Convenience	211	[38]	Hernandez, 2013	HPV	211
2010	National	Community based	17–87	Random + Convenience	807	[40]	Lau, 2012a	Leptospirosis	807
						[41]	Lau, 2012b	Leptospirosis	807
						[33]	Duncombe, 2013	Dengue	794
						[19]	Lau, 2014	LF	807
						[20]	Lau, 2016a	LF	376
						[43]	Lau, 2016b	Rickettsia*	197
						[31]	Coutts, 2017	LF	807
						[34]	Lau, 2017b	RRV	196

2010	Sub-national	Employees of the LBJTMC	19–71	Census	231	[39]	Ly, 2014	HBV	231
2011	Sub-national	Students form elementary school and community college	4–35	Census	723	[37]	Mahamud, 2014	VZV	723
2011	National	School based (TAS-1)	5–10	Systematic	1134	[18]	Chu, 2013	LF	949
						[21]	Won, 2018	LF	937
						[27]	Restrepo, 2022	LF	937
2014	National	Targeted populations: adult workers, community members, School children	2–≥65	Convenience + random	1132	[30]	Lau, 2017a	LF	1078
						[24]	Graves, 2020	LF	670
2015	National	School based (TAS-2)	5–10	Systematic	768	[21]	Won, 2018	LF	768
						[27]	Restrepo, 2022	LF	768
2016	National	School based (TAS-3)	5–10	Systematic	1143	[22]	Sheel, 2018	LF	1143
						[23]	Lau,2020a	LF	1143
						[27]	Restrepo, 2022	LF	1143
2016	National	Community based	8–93	Random	2671	[22]	Sheel, 2018	LF	2496
						[23]	Lau, 2020a	LF	2496
						[28]	Lemin, 2022	LF	2671
						[29]	Wangdi, 2022	LF	2671
						[32]	Restrepo, 2023	LF	2671
2016-2017	National	Pregnant women attending clinic prenatal services	NR	Convenience	277	[35]	Hancock, 2017	Zika	277
2017	National	Household members of positive cases	<1–94	Convenience	226	[36]	Sharp, 2023	Dengue	226
2019	National	Community based	≥5	NR	2081	[26]	Hast, 2020	LF	2081
**Samoa**									
1999-2000	Sub-national	Pregnant women attending hospital prenatal services	15–48	Convenience	441	[53]	Sullivan, 2004	HIV	441
2002	NR	NR	NR	NR	1289	[16]	Armstrong, 2006	HCV	1289
2004-2005	National	Pregnant women attending clinic prenatal services	15–44	Convenience	300	[54]	MoH/WHO, 2006	HIV	299
						[56]	Cliffe, 2008	HIV	299
2004-2005	National	General population attending an STI clinic	16–43	Convenience	101	[54]	MoH/WHO, 2006	HIV	101

2004	National	Community based	NR	Random	12719	[44]	Huppatz, 2009	LF	12719
2007	Sub-National	Community based	≥5	Random clustered	6648	[47]	Joseph, 2011a	LF	6648
2008	National	Pregnant women attending clinic prenatal services	15–49	Convenience	298	[55]	MoH/WHO, 2008	HIV/HBV	298
2008	Sub-national	Community based	2–92	Census/ Random	2474	[46]	Joseph, 2011b	LF	2474
						[45]	Joseph, 2011C	LF	2474
2018	National	Community based	≥5	Random + convenience	4420	[48]	Willis, 2020	LF	3852
						[49]	Lau, 2020b	LF	3852
						[50]	Mayfield, 2020	LF	2322
						[52]	Willis, 2023	Scabies	2868
2018	Sub-national	School based (rural areas)	4–15	Census	833	[51]	Taiaroa, 2021	Scabies	833
2019	National	Community based	≥5	Random	2796	[50]	Mayfield, 2020	LF	2594
						[52]	Willis, 2023	Scabies	2796

NR- not reported; STH – soil-transmitted helminthiasis; HCV- hepatitis C virus; LF – lymphatic filariasis; HPV – human papillomavirus; RRV- Ross River virus; LBJTMC - e Lyndon B. Johnson Tropical Medical Center; HBV- hepatitis B virus; VZV- varicella-zoster virus; TAS – transmission assessment survey; HIV – human immunodeficiency virus; STI – sexually transmitted illness. **Rickettsia spp., Bartonella spp., Ehrlichia spp., Coxiella burnetii.*

Supplementary Table S2. Publications included in the systematic review reporting data from sources other than surveys (national reports and summary of multiple studies).

Year	Geographical level	Country	Disease	Ref	Author, year
2017	National	Samoa	HBV, HCV	[57]	MoH, 2018
2018	National	Samoa	HBV, HCV	[58]	MoH, 2019
1999-2005	National	American Samoa and Samoa	LF	[14]	WHO, 2006
1998-2017	National	Samoa	LF	[15]	Graves, 2021

HBV- hepatitis B virus, HCV- hepatitis C virus, LF- lymphatic filariasis, MoH- Ministry of Health. WHO – World Health Organization.

S2.2. Reported Prevalence

Supplementary Table S3. Samoan islands' lymphatic filariasis prevalence reported in publications, between 2000–2023 by year[1].

Year	Reporting level	Study design	Sampling design	Antigen				Antibodies							Microfilariae slides			Ref
									Wb123		Bm14		Bm33					
				Test	N. tested	Positive	Rate	N. tested	Positive	Rate	Positive	Rate	Positive	Rate	N. tested	Positive	Rate	
**American Samoa**																		
1999	National	Baseline survey	Convenience sampling	ICT	3018	498	16.5	-	-	-	-	-	-	-	-	-	-	[14]
2001	Overall	Sentinel sites	Convenience sample	ICT	1024	118	11.52	-	-	-	-	-	-	-	118	28	2.73	[17]
	Aunu'u						0.6	-	-	-	-	-	-	-	-	-	-	[17]
	Faga'itua						20.5	-	-	-	-	-	-	-	-	-	-	[17]
	Fagasa						11	-	-	-	-	-	-	-	-	-	-	[17]
	Pago Pago						10.7	-	-	-	-	-	-	-	-	-	-	[17]
2003	Overall	Sentinel sites	Convenience sample	ICT	917	126	13.74	-	-	-	-	-	-	-	126	9	0.98	[17]
	Aunu'u						7.6	-	-	-	-	-	-	-	-	-	-	[17]
	Faga'itua						21.7	-	-	-	-	-	-	-	-	-	-	[17]
	Fagasa						15.7	-	-	-	-	-	-	-	-	-	-	[17]
	Pago Pago						7.8	-	-	-	-	-	-	-	-	-	-	[17]
2006	Overall	Sentinel sites	Convenience sample	ICT	1371	13	0.95	-	-	-	-	-	-	-	13	2	0.15	[17]
	Aunu'u						0	-	-	-	-	-	-	-	-	-	-	[17]
	Faga'itua						1.6	-	-	-	-	-	-	-	-	-	-	[17]
	Fagasa						0.3	-	-	-	-	-	-	-	-	-	-	[17]
	Pago Pago						0.6	-	-	-	-	-	-	-	-	-	-	[17]
	Vaitogi	Spot check					2.1	-	-	-	-	-	-	-	-	-	-	[17]
	National	Community-based	Census sampling	ICT	579	24	4.2	565	-	-	76	13.5	-	-	24	1	0.2	[25]
	Afao				160	7	4.4	152	-	-	19	12.5	-	-	7	1	0.6	[25]
	Asili				191	7	3.7	172	-	-	25	14.5	-	-	7	0	0	[25]
	Seetaga				218	10	4.6	214	-	-	32	14.9	-	-	10	0	0	[25]
2007	National	C-Survey	Simple random	ICT	1881	43	2.29 (1.66-3.07)	-	-	-	-	-	-	-	43	5	0.27 (0.09-0.62)	[31]
2010	National	Community-based	Random sampling (Tutuila) and convenience sampling	Og4C3 (cutoff titre >128)	805	6	0.7% (0.3-1.6)	807	65	8.1 (6.3-10.2)	144	17.9 (15.3-20.7)	-	-	-	-	-	[19,20]
	National	Community-based		Og4C3 (cutoff tit>32)	805	26	3.2 (0.6-4.7)	-	-	8.1	-	17.9	-	-	-	-	-	[19,20]
2011	National	TAS-1	Systematic sampling of targeted population	ICT	937	2	0.2 (0-0.8)	1112	11	1.0 (0.5-7.6)	76	6.8 (5.4-8.5)	133	12.0 (10.1-14.0)	2	0	0	[18,21,27]

2014	Tutuila	Adult workers	Targeted population	ICT/Og4C3	670	12	1.8	666	75	11.3	79	11.9	-	-	8	1	0.15	[30]
	National	School-based			337	3	0.89	-	-	-	-	-	-	-		1	0.3	[30]
	National	Community-based			795	26	3.27	795								3	0.37	[30]
	Ili'ili, Vaitogi, Futiga				418	10	2.4	112	-	18.8 (11.4-28.5)	-	12.5 (7.1-21.1)	-				-	[30]
	Fagali'i				58	10	17.2	38	-	55.2 (39.6-69.8)	-	43.4 (32.4-55.0)	-	-	-	-	-	[30]
	out of suspected hotspots				602	8	1.5	598	-	10.5 (7.6-14.3)	-	9.6 (7.5-12.3)	-	-	-	-	-	[30]
2015	National	TAS-2	Systematic sampling	FTS	768	1	0.1 (0-0.7)	836	30	3.6 (2.4-5.1)	25	3 (2.0-4.4)	65	7.8 (6.1-9.8)	-	-	-	21,27]
2016	National	TAS-3 – School-based	Systematic sampling	FTS	(1143) 1143	9	0.8 (0.4-1.5)	1139	94	8.3 (6.7-10.0)	18	1.6 (0.9-2.5)	25	20.8 (18.5-23.3)	9	1	11.1 (0.09)	[22,23,27]
	National	Community-based	Stratified random cluster	FTS	(2496) 2507	102	4.1 (6.2; 4.5-8.6)	2507	-	27.9 (24.6-31.4)	-	13.9 (11.2-17.2)	-	47.3 (42.1-52.6)	86	22	25.6 (0.9)	[22,23,28,29,32]
	National				(2671) 2710	135	5.1 (4.25-5.95)	2671	684	25.6 (23.96-27.31)	350	13.1 (11.85-14.41)	1219	45.9 (43.73-47.45)	114	34	1.3 (0.88-1.80)	[22,23,28,29,32]
2019	National	Community-based	Systematic random sampling	CFA4	2081	47	2.7	-	-	-	-	-	-	-	-	-	-	[26]
**Samoa**																		
1999	National	Baseline survey	Convenience sample	ICT	7006	317	4.5											
2000	National	Sentinel sites	Convenience sample	ICT	676	55	8.1								88	7	8	[14]
2001	National (4 villages)	Sentinel sites	Convenience sample	ICT	1392	67	4.8								67	10	14.9	[14]
2002	National (10 villages)	Sentinel sites	Convenience sample	ICT	2141	96	4.5								2265	6	0.3	[14]

2003	National (6 villages)	Sentinel sites	Convenience sample	ICT	881	14	1.6								881	6	0.7	[14,15]
2004	National	Community-based	Convenience sample	ICT	12719	48	1.1								12719	55	0.4	[15,54,56]
	AUA				621		0.2										0	[15,54,56]
	NWU				1480		2.9										1.4	[15,54,56]
	ROU				5664		1										0.2	[15,54,56]
	SAV				4907		0.9										0.3	[15,54,56]
2007	National	Community-survey (C-survey)	Targeted (*16) Stratified random cluster	ICT	6448		2.5			30.7							0.6	[15,47]
	AUA			CFA	415	-	1			71							0	[15,47]
	NWU				1549	-	5.2			40							1.4	[15,47]
	ROU				1746	-	2.7			25							0.6	[15,47]
	SAV				2738	-	1.3			44							0.2	[15,47]
2008	National	Spot check survey		ICT	2474													[15,45,46]
	Tafua (Savai'i)	Community-based	Census sampling + population representative)	Og4C3	344	-	8.4 (5.7-11.9)			34.3 (29.3-39.6)							0.6 (0.1-2.1)	[15,45,46]
	Pauapua (Savai'i)				448	-	2.5 (1.2-4.4)			ND							0 (0.0-0.8)	[15,45,46]
	Fasitoo-Tai (Upolu)				617	-	14.6 (11.9-17.6)			74.9 (71.3-78.3)							3.2 (2.0-5.0)	[15,45,46]
	Siufaga (Upolu)				495	-	5.1 (3.4-7.2)			64.9 (60.8-68.8)							0 (0.0-0.7)	[15,45,46]
	Falefa (Upolu)				570	-	1.6 (0.7-3.2)			64.8 (60.5-69.1)							0 (0.0-0.7)	[15,45,46]
2013	National	TAS-1		ICT	3585	25	0.7											[15]
	NWU				1271	19	1.5											[15]
	SAV				1098	5	0.5											[15]
	AUA + ROU				1216	1	0.1											[15]
2017	National	TAS-2		ICT	3383	133	3.9											[15]
	NWU				1031	70	6.8											[15]
	SAV				1059	45	4.3											[15]
	AUA + ROU				1263	18	1.4											[15]
2018	National	Community-based	Population representative cluster survey	FTS	(3852) 4420	122	4.3 (3.5-5.2)								121	13	1.7	[15,48-50]
	AUA				741		3.5 (2.1-5.7)											[15,48-50]
	NWU				645		6.2 (4.9-7.9)											[15,48-50]
	ROU				323		1.8 (1.0-3.4)											[15,48-50]
	SAV				325		3.3 (2.0-5.6)											[15,48-50]
2019	National	Community-based	Stratified clustered	FTS	2594		4.7 (4.0-5.6)											[50]

1 – Discrepancies in sample size and study design are shown in Supplementary Table S1.

Supplementary Table S4. Samoan islands' NTDs, VPDs, VBDs, BBVs and zoonoses prevalence reported in publications, between 2000–2023 by year.

	Year	Level	Study design	Sampling design	Diagnostic test	N tested	Positive	Rate	Ref
**American Samoa**									
**Soil Transmitted Helminths**	**2001/02**	National	School based	Convenience	Kato Katz stool microscopy	60	2	3.3%	[16]
**Dengue**	**2010**	National	Community-based	Random + Convenience	IgG anti-DENV (ELISA)	974	759	95.6%	[33]
	**2017**	National	Household members of acute confirmed cases	Convenience	IgM anti-DENV (ELISA) and RT-PCR	226	27	3.1%	[36]
**Zika**	**2016/17**	National	Pregnant women attending clinic prenatal services	Convenience	IgM anti-ZiKa and PCR	277	86 (IgM)<br>0 (PCR)	31.0%	[35]
**RRV**	**2010**	National	Community based	Random + Convenience	IgG anti-RRV	196	145	74.0%	[34]
**HPV**	**2008/09**	Sub-national	Community and hospital based	Convenience	RT-PCR	211	21	10.0%	[38]
**VZV**	**2011**	Sub-national	School based	Census	IgG anti-VZV	715	594	83.1%	[37]
**HBV**	**2010**	Sub-national	Hospital workers	Census	HbsAg	231	6	2.6%	[39]
**HCV**	**2002**	NR	NR	NR	Anti-HCV (enzyme immunoassay)	1289	1	0.16%	[59]
**Leptospirosis**	**2004**	National	Population representative	Random	MAT	341	58	17.0%	[41,42]
	**2010**	National	Community based	Random + Convenience	MAT	807	125	15.5%	[40,41]
**Rickettsia spp., Bartonella spp., Ehrlichia spp. and Coxiella burnetii**	**2010**	National	Community based	Random + Convenience	IgG (ELISA)	197	0	0%	[43]

(cont.)

	Year	Level	Study design	Sampling design	Diagnostic test	N tested	Positive	Rate	Ref
**Samoa**									
**Scabies**	**2018**	National	Community based	Random+ Convenience	Clinical examination			3.0%	[52]
**Scabies**	**2018**	Sub-national	School-based (rural areas)	Census	Clinical examination	833	120	14.4%	[51]
**Scabies**	**2019**	National	Community based	Random+ Convenience	Clinical examination			4.2%	[52]
**HIV**	**2001**	Sub-national	Pregnant women	Convenience	Imx HIV1/2 + WB	441	0	0%	[53]
**HIV**	**2004/05**	National	Pregnant women	Convenience	Determine and Serotia HIV test + WB	299	0	0%	[54]
**HIV**	**2004/05**		General population attending a STI clinic	Convenience	Determine and Serotia HIV test + WB	101	0	0%	[54]
**HIV**	**2008**		Pregnant women	Convenience		298	0	0%	[55]
**HBV**	**2008**		Pregnant women	Convenience	HbsAg (Determine and Serotia)	298	32	10.7 (7.6-15.1%)	[55]
**HBV**	**2017**		Pregnant women	Census	NR	4386	59	1.35%	[57]
**HBV**	**2017**		Immigration screening (workers leaving + entering country)	Census	NR	1737	39	2.25%	[57]
**HBV**	**2017**		Blood donor screening	Census	NR	1382	43	3.11%	[57]
**HBV**	**2018**		Pregnant women	Census	NR	4815	56	1.16%	[58]
**HBV**	**2018**		Immigration screening (workers leaving + entering country)	Census	NR	1813	38	2.10%	[58]
**HBV**	**2018**		Blood donor screening	Census	NR	3041	71	2.33%	[58]
**HCV**	**2002**	NR	NR	NR	Anti-HCV (enzyme immunoassay)	1359	1	0.08%	[59]
**HCV**	**2017**		Pregnant women	Census	NR	3012	0	0%	[57]
**HCV**	**2017**		Immigration screening (workers leaving + entering country)	Census	NR	1609	1	0.06%	[57]
**HCV**	**2017**		Blood donor screening	Census	NR	1379	1	0.07%	[57]
**HCV**	**2018**		Pregnant women	Census	NR	4266	2	0.05%	[58]
**HCV**	**2018**		Immigration screening (workers leaving + entering country)	Census	NR	1608	0	0.0%	[58]
**HCV**	**2018**		Blood donor screening	Census	NR	2958	4	0.14%	[58]
